# Classification, quantification, and thermotolerance assessment of lactic acid bacteria in yogurt using bacterial melting curve analysis

**DOI:** 10.3389/fmicb.2026.1751797

**Published:** 2026-03-04

**Authors:** Yi Liang, Haotang Wei, Feng Chen, Jialei Wang, Shengbin He, Quanzhi Chen, Zhao Li

**Affiliations:** 1 The Second Nanning People’s Hospital, Nanning, China; 2 University Engineering Research Center of Advanced Technologies in Medical and Biological Intelligent Manufacturing, Guangxi Colleges and Universities Key Laboratory of Biological Molecular Medicine Research, School of Basic Medical Sciences, Guangxi Medical University, Nanning, Guangxi, China

**Keywords:** identification, lactic acid bacteria, melting curve, quantitative detection, SYTO 9

## Abstract

**Introduction:**

The expanding market for probiotic fermented foods like yogurt necessitates advanced methodologies to validate product claims. While multiple analytical approaches exist for characterizing LAB, most of them are either costly or inefficient.

**Methods:**

Here, for the first time, we put forward a bacterial melting curve analysis (BMCA) method to classify lactic acid bacteria (LAB) rapidly. This approach employs SYTO 9 and a thermoregulated fluorometer to simultaneously monitor LAB viability and double-stranded DNA (dsDNA) content. Ramping the temperature from physiological (37°C) to denaturing (98°C) levels allowed the concurrent recording of bacterial inactivation kinetics and dsDNA dissociation profiles, yielding distinct species-specific signatures.

**Results:**

Validation using three reference strains demonstrated clear differentiation of LAB via multivariate dimensionality reduction of the melting curves. The method was successfully applied to classify and quantify LAB in yogurt without using antibodies. Furthermore, BMCA was utilized to assess the thermotolerance of LAB in different formulations, revealing enhanced thermal stability when the bacteria were encapsulated within calcium alginate gel particles.

**Conclusion:**

The BMCA enabled LAB identification and measurement in yogurt samples without requiring antibody-based techniques. Additionally, the BMCA method can also be employed to evaluate thermal stability of LAB in formulations.

## 1 Introduction

Growing awareness of probiotics’ health benefits has led to the heightened consumers focus on functional foods containing these microorganisms. LAB represent the predominant microbial species utilized as probiotics in functional food applications (Calvo et al., 2025; Liu et al., 2025). These microorganisms are routinely incorporated into fermented dairy products including yogurt and cultured milk beverages to impart gastrointestinal health benefits (Grzelak-Blaszczyk et al., 2023; Liu et al., 2024; Shan et al., 2024). For optimal physiological effects, probiotic strains should maintain metabolic activity, species diversity, and sufficient population density throughout product shelf life (Shi et al., 2022; Hinojosa-Avila et al., 2025). Consequently, commercial products marketed with probiotic health claims must demonstrate compliance with these diversity and viability standards through rigorous quality control measures.

While multiple analytical approaches exist for characterizing LAB, conventional culture-based techniques remain the most widely used ones due to their simpleness and low-cost testing. However, various LAB species are cultivated under similar conditions, impeding the quick and accurate determination of the viable count of each species via the cultivation methods (Suele et al., 2014; Shi et al., 2022). With specific primers, polymerase chain reaction (PCR) is becoming more and more popular for determining LAB qualitatively and quantitatively, which unfortunately cannot distinguish between dead and live LAB (Li et al., 2022; Traylor et al., 2024; Zang et al., 2025). Nascent techniques for the characterization of LAB include flow cytometry (He et al., 2017; Yolmeh et al., 2024), 16S rRNA gene sequencing (Loughrin and Agga, 2025; Yang et al., 2025), surface-enhanced Raman scattering (Shang et al., 2023; Wu H. et al., 2025), mass spectrometry (Valletta et al., 2023; Kim et al., 2024), colorimetric analysis (Chen et al., 2024a; Zhang et al., 2024)，and metagenomic (Kushargina et al., 2024; Vermote et al., 2025). These techniques required either large equipments, high-cost reagents, trained operators, or a long testing time. In addition, few of them could achieve the simultaneous classification and quantification of multiple LAB.

SYTO 9‌ is a fluorescent dye that ‌targets dsDNA‌, exhibiting ‌weak fluorescence in its unbound state‌ and ‌strong fluorescence upon binding dsDNA‌. This characteristic facilitates real-time tracking of DNA synthesis and melting kinetics in qPCR assays. As a biomembrane permeable dye, SYTO 9 is also widely used in dsDNA staining of bacteria, labeling all the bacterial cells green fluorescence regardless of their viability status (He et al., 2020; He et al., 2024; Zheng et al., 2025). In an effort to establish a rapid method for LAB detection, here we unexpectedly found that although SYTO 9 exhibits non-discriminatory staining across LAB viability states, the pronounced fluorescence enhancement occurs exclusively in dead LAB cells due to the compromised membrane barrier function. We assumed that such characteristics enable the SYTO 9 to be used for tracing simultaneously the viability (via biomembrane integrity) and dsDNA content of LAB. Based on the significant inter-species variations in thermotolerance and genomic composition, we further coupled the SYTO 9 with a thermoregulated fluorometer to establish a BMCA method for LAB classification. The multifunctional BMCA platform not only exhibited good practicability in the classification and quantification of multiple live LAB in yogurt, but also achieved the rapid evaluation of thermotolerance of the LAB in different formulations.

## 2 Experimental section

### 2.1 Reagents and lactic acid bacteria strains

SYTO 9 fluorescent dye was acquired from Thermo Fisher Scientific (Waltham, MA, United States). *Streptococcus thermophilus* BH16 (*S. thermophilus*), *Lactobacillus casei* ATCC344 (*L. casei*), and *Lactobacillus bulgaricus* Y7135 (*L. bulgaricus*) cultures were maintained in our laboratory stock collections. Calcium chloride (CaCl_2_), sodium alginate, and De Man, Rogosa, and Sharpe (MRS) broth were procured from Baisi Biotechnology Co., Ltd. (Hangzhou, Zhejiang, China) and Aladdin Bio-Chem Technology Co., Ltd. (Shanghai, China), respectively. Additional chemical reagents were sourced from Sinopharm Chemical Reagent Co., Ltd. (Shanghai, China). Microbiological-grade water, prepared using a Milli-*Q* RG purification system and sterilized by filtration through a 0.22 μm membrane filter, was employed for all buffer solutions.

### 2.2 Investigation on the selectivity of SYTO 9 against different lactic acid bacteria

For all the three different live LAB strains, a 100 μL aliquot of LAB suspension (about 1 × 10^8^ CFU/mL in PBS) was pipetted into a 1.5 mL Eppendorf tube, followed by adding 100 μL SYTO 9 solution (1 μM/mL). The solution was incubated at room temperature for 5 min, which was then subjected to fluorescence spectral scanning by a fluorescence spectrophotometer. The excitation wavelength was set to be 450 nm and integration time was 3 s. Fluorescence intensity at 520 nm was recorded and used for calculating the mean fluorescence intensity. To cause LAB cells death, the LAB suspensions were heated at 80 °C for 10 min. The dead LAB cells were stained and analyzed as the steps above.

The stained LAB samples were also analyzed by fluorescence microscope (DMi8, Leica, Germany) and flow cytometry (FCM, Accui C5, BD, United States) as described previously (He et al., 2024). For FCM analysis, specifically, a laser of 20 mW at 488 nm was used for excitation. The optical configuration of the flow cytometer included a 530/30 nm bandpass filter to detect green fluorescence from SYTO 9. Data were acquired in log-scale, with the acquisition threshold set on the forward scatter (FSC) signal. Photomultiplier tube voltages were configured at 350 V for FSC and 200 V for the green fluorescence channel (FL1). A dual-parameter gate based on FSC and FL1 intensities was applied to distinguish bacterial cells from background noise. For statistical reliability, a minimum of 5,000 cellular events were recorded per sample. Subsequent data analysis was performed using FlowJo software (BD, United States) to determine precise fluorescence intensity.

### 2.3 Preparation of lactic acid bacteria

*Streptococcus thermophilus*, *L. casei*, and *L. bulgaricus* were cultivated in MRS broth using established protocols described by He et al. (2017) and Chen et al. (2024a). Upon reaching mid-exponential phase (OD_600_ value reached about 1.0), cellular biomass was harvested via centrifugation (5,000 × g, 5 min). The pellets underwent three successive washes with sterile phosphate-buffered saline (PBS, containing 137 mM NaCl, 2.7 mM KCl, 10 mM Na_2_HPO_4_ and 1.8 mM KH_2_PO_4_, at pH 7.4). Then the LAB cells were resuspended in fresh PBS to achieve a standardized optical density of OD_600_ = 0.5. Cell viability exceeding 99% was confirmed for all processed suspensions using the SYTO 9/propidium iodide (PI) dual-staining assay as described previously (Chen et al., 2024a).

### 2.4 Heat treatment on the viability of lactic acid bacteria

Bacterial suspensions (1 × 10^7^ to 1 × 10^8^ CFU/mL) were allocated to eight experimental cohorts. Each cohort underwent thermal exposure in a water bath at distinct temperatures (35, 40, 45, 50, 55, 60, 65, and 70 °C) for 60 s to assess thermotolerance. Immediately following heat treatment, aliquots were aseptically collected for viability quantification via standardized plate counts as described previously (Wang et al., 2023; Cao et al., 2025). Serial decimal dilutions were prepared, and 100 μL aliquots were plated in triplicate onto sterile MRS agar. After 72 h aerobic incubation at optimal growth temperatures (37 °C), colonies were enumerated on plates exhibiting 30–300 colonies per plate.

### 2.5 Melting curve analysis for the classification and quantification of lactic acid bacteria

To classify different LAB strains, a 15 μL bacterial suspension (1 × 10^6^–1 × 10^8^ CFU/mL) was mixed with an equal volume of 1 μM SYTO 9 solution in PCR tubes. Samples were analyzed using a thermoregulated fluorometer (StepOnePlus™, Thermo Fisher Scientific, United States) with the following thermal profile: 5 s at 37 °C, ramp to 98 °C at 0.1 °C/s, and 5 s at 98 °C. The fluorescence intensity and temperature were used to plot melting curves, which were subsequently processed through multifactor dimensionality reduction via the software SPSSPRO analytics platform.^1^

For the simultaneous classification and quantification of LAB in yogurt, the yogurt sampled from the grocery store was directly subjected to 10 times gradient dilution by PBS, with a final dilution of 0 to 1 × 10^2^ CFU/mL. A 5 μL aliquot of bacterial suspension was pipetted into a PCR tube. Another 5 μL aliquot of SYTO 9 solution (2 μM) and a 10 μL aliquot of MRS medium (2×) were added to the tube. Such protocol ensured that each tube contained no more than one LAB cell. The LAB containing tubes were incubated at 37 °C for 12 h to amplify the LAB. After amplification, the tubes were placed in a thermoregulated fluorometer for testing as described above.

### 2.6 Thermotolerance assessment of LAB in three different formulations

A 500 μL aliquot of LAB solution (1 × 10^8^ CFU/mL) was mixed with 500 μL calcium chloride (CaCl_2_, with final concentrations ranging from 0 to 50 mg/mL), or sodium alginate (with final concentrations ranging from 0 to 200 mg/mL). The mixtures were incubated 10 min, and then a 15 μL LAB suspension was mixed with an equal volume of 1 μM SYTO 9 solution in PCR tubes. The tubes were placed in a thermoregulated fluorometer for melting curve analysis as described above. To prepare alginate gel embedded LAB, 1 mL LAB suspension (1 × 10^8^ CFU/mL) was mixed homogenously with 1 mL alginate solution (20 mg/mL), as described previously (Cabrita et al., 2013; He et al., 2014). The mixture was added into 1% calcium solution by peristaltic pump and left to harden for 2 h. The alginate beads containing LAB were directly subjected to melting curve analysis as described Section 2.5.

For all the experiments, the mean values and standard deviations (SD) were calculated by Origin software. All the difference analyses were performed using the Student’s *t*-test.

## 3 Results and discussion

### 3.1 Work principle of bacterial melting curve analysis

**Scheme 1:**
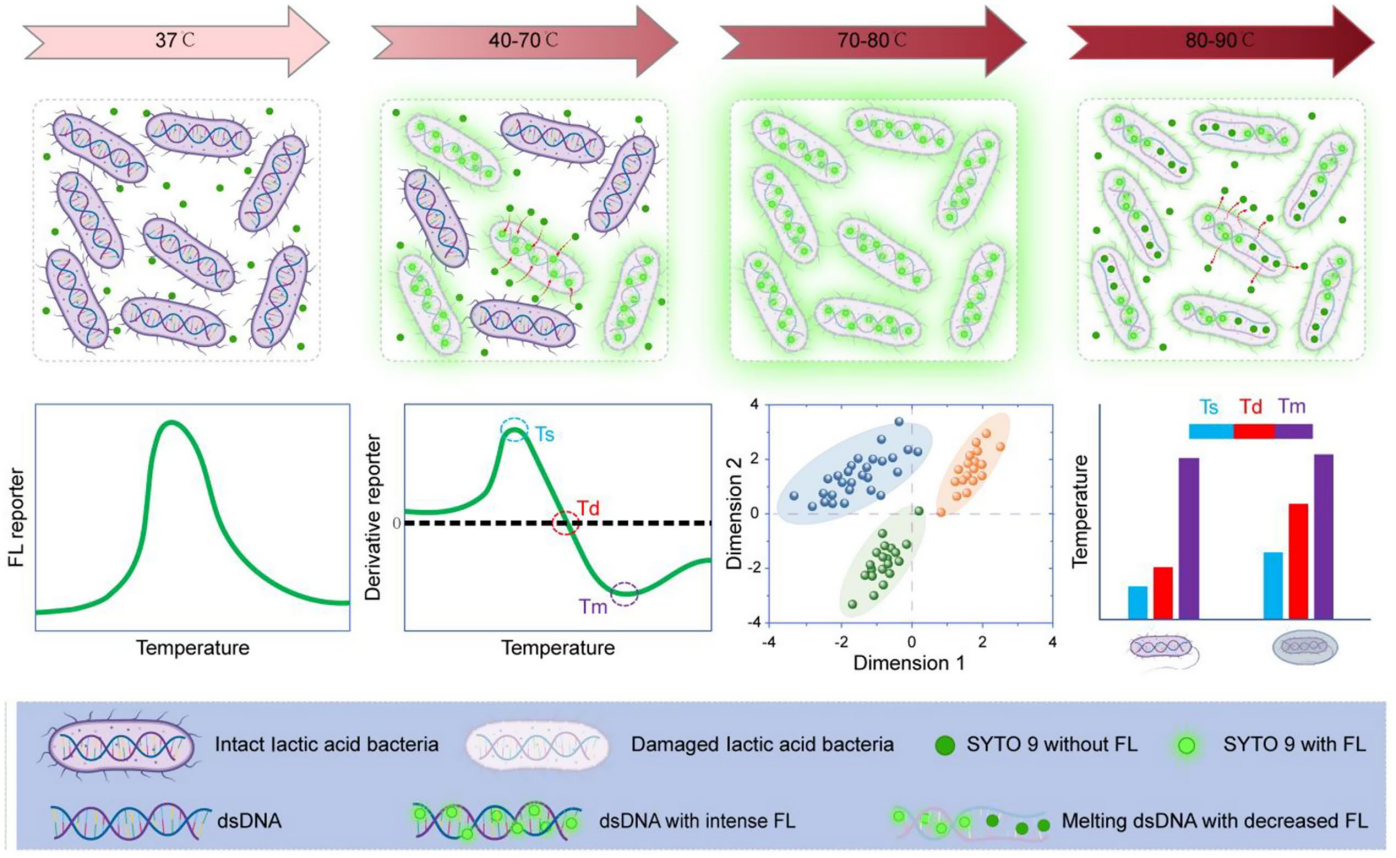
Principle of bacterial melting curve for live LAB analysis.

Throughout the course of evolutionary biology, microbial lifeforms like bacteria have developed extensive genetic variation within their DNA sequences as an adaptive strategy against dynamic environmental challenges. Such genomic variability fundamentally drives metabolic and morphological differentiation, enabling the emergence of diverse bacterial variants characterized by distinct thermal tolerance profiles, antimicrobial resistance patterns, and cellular membrane properties (Chen et al., 2024b; Letizia et al., 2025). This biological principle provides a potential approach for distinguishing LAB based on their differences in thermotolerance and DNA composition. Scheme 1 describes the work principle of melting curve for thermotolerance and DNA composition analysis of LAB. The membrane semipermeable and dsDNA-specific dye, SYTO 9, was incubated with LAB cells. As the temperature rose from 40–70 °C, progressive membrane disruption led to LAB cell death, facilitating the influx of SYTO 9 into the cells and enhancing fluorescence intensity. However, further heating caused gradual denaturation of bacterial genomic dsDNA, leading to a subsequent decline in fluorescence signal. Variations in thermostability and dsDNA composition among different LAB strains enabled their differentiation by analyzing the melting profiles using multidimensional factor reduction techniques. Meanwhile, the three characteristic values, semilethal temperature (Ts), total lethal temperature (Td), and DNA melting temperature (Tm) can be captured for assessing the thermotolerance of a LAB strain. The selection of SYTO 9 was based on its dual specificity: an affinity for dsDNA and a selective staining of dead bacterial cells. This dual specificity enables the generation of distinctive bacterial melting curves. In contrast, other nucleic acid stains, such as PI and ethidium bromide (EB), do not possess these combined properties.

### 3.2 The selectivity of SYTO 9 against lactic acid bacteria of different viability

**Figure 1:**
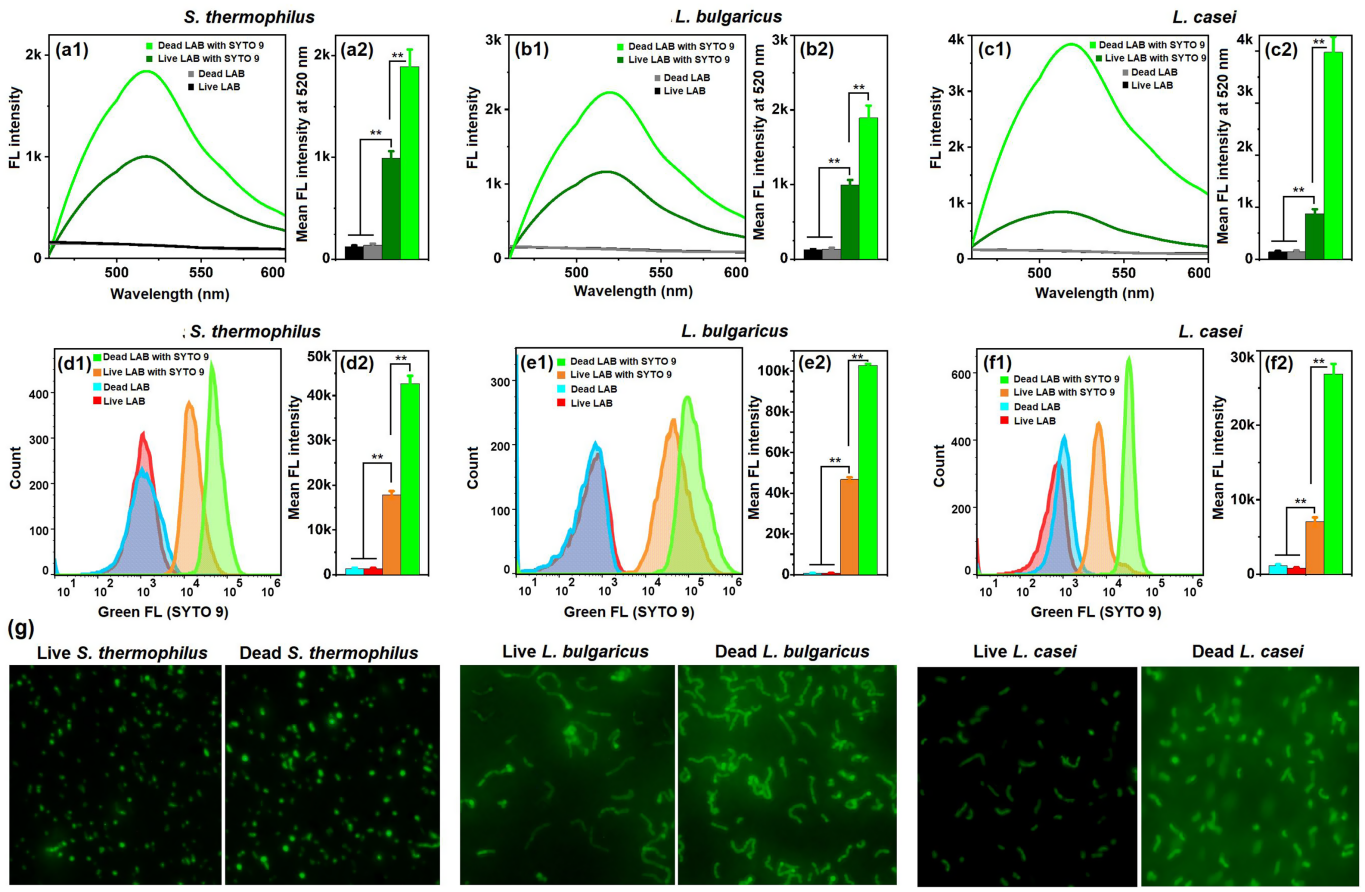
Selectivity of SYTO 9 against LAB of different viability. **(a1–c1**) Representative fluorescence (FL) spectra of live/dead *S. thermophilus* **(a1)**, *L. bulgaricus* **(b1)**, and *L. casei* **(c1)** in the presence and absence of SYTO 9. **(a2–c2)** Mean FL intensity at 520 nm of the above samples. **(d1–f1)** Representative FCM histogram of live/dead *S. thermophilus* **(d1)**, *L. bulgaricus* **(e1)**, and *L. casei* **(f1)** in the presence and absence of SYTO 9. **(d2–f2)** Mean FL intensity of the above samples. Five independent experiments were conducted, and data are represented as mean ± SD (*n* = 5). The difference was significant, as indicated by one asterisk for *p* < 0.05 and two asterisks for *p* < 0.01. **(g)** Fluorescence microscope observation of live/dead *S. thermophilus*, *L. bulgaricus*, and *L. casei* in the presence of SYTO 9.

Although SYTO 9 is known for its good permeability to live cells and bacteria, here we found by chance that SYTO 9 exhibited greater permeability to dead *S. thermophilus* than to live *S. thermophilus*. To further extend this phenomenon to other LAB strains, we used *S. thermophilus*, *L. bulgaricus*, and *L. casei* as LAB models to systematacially investigate the selectivity of SYTO 9 against live and dead LAB. These three LAB strains were chosen because they are the most widely used in dairy industry in China. Figures 1a–c shows the fluorescence (FL) spectra of live and dead cells for the three LAB strains. The LAB were incubated with SYTO 9 at a final concentration of 1 μM/mL and with free SYTO 9 molecules remaining in the LAB solutions. Similar to the previously reporters, the SYTO 9 could enter live LAB and showed significant FL enhancement at 520 nm once binding with dsDNA of the LAB. However, we found that for all the three LAB strains, a further pronounced FL enhancement occurs in dead LAB cells. The degree of this enhancement varied among strains; among them, *L. casei* exhibited the greatest difference in FL intensity at 520 nm between dead and live cells. The results of quantitative FCM analysis and semi-quantitative fluorescence microscope observation agreed well with that of FL spectra (Figures 1d–g). Therefore, it is supposed that although SYTO 9 exhibits non-discriminatory staining across LAB viability states, the pronounced fluorescence enhancement occurs exclusively in dead LAB cells due to the compromised membrane barrier function.

### 3.3 The species-specific bacterial melting curves were shapes by two sequential thermal transitions

SYTO 9 is also a selective dye for dsDNA. It generates green emission upon intercalation into the dsDNA helix and this signal diminishes when thermal denaturation separates the DNA strands, releasing unbound dye molecules. Based on this mechanism, the dye has become a staple tool in quantitative PCR (qPCR), serving both to monitor real-time DNA amplification and to characterize products via melting curve profiling (Horakova et al., 2011; Wan et al., 2016; Shen et al., 2023). Given that thermotolerance and genomic dsDNA exhibit variations across LAB species and strains, we explored the possibility of using SYTO 9 combined with a thermoregulated fluorometer to classify LAB through melting curve profiling.

**Figure 2:**
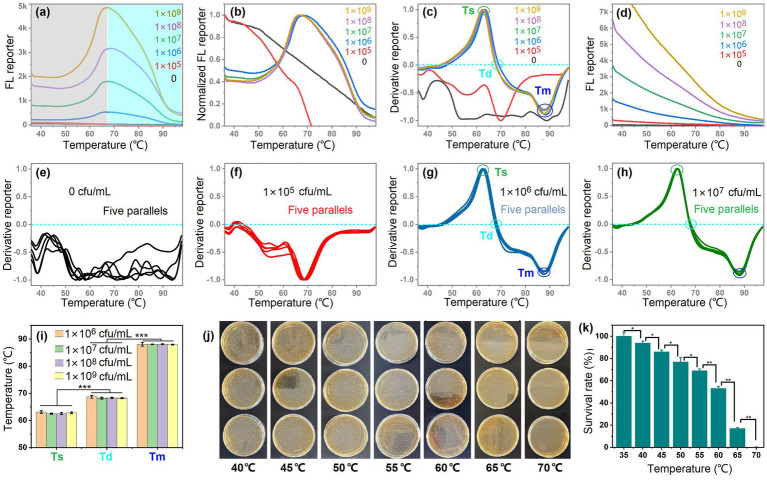
Two sequential thermal transitions shaped the bacterial melting curves. **(a)** Melting curves for live *S. thermophilus* of different concentrations, ranging from 0 to 1 × 10^9^ CFU/mL. **(b,c)** Normalization **(b)** and derivation **(c)** of the FL reporter for the above samples. **(d)** Melting curves for dead *S. thermophilus* of different concentrations. **(e–h)** Melting curves for repeated experiments of live *S. thermophilus* of different concentrations. **(i)** Statistic analysis of semi-lethal temperature (Ts), total lethal temperature (Td), and DNA melting temperature (Tm) of the above samples. Five independent experiments were conducted, and data are represented as mean ± SD (*n* = 5). The difference was significant, as indicated by three asterisks for *p* < 0.001. **(j)** Influence of temperature on the viability of *S. thermophilus* evaluated by plate counting. **(k)** Survival rate of the LAB calculated from the plate counting results. Three independent experiments were conducted, and data are represented as mean ± SD (*n* = 3). The difference was significant, as indicated by one asterisk for *p* < 0.05 and two asterisks for *p* < 0.01.

Figure 2a displays the dynamic FL (*λ* = 520 nm) changes for *S. thermophilus* of different concentrations observed during the LAB cells heating from 37 °C to 98 °C. The FL intensity correlated positively with LAB concentration. When the LAB concentration exceeded 1 × 10^6^ CFU/mL, the curve profiles remained consistent across varying concentrations. Normalization of FL signals resulted in nearly superimposable curves (Figure 2b), suggesting that the observed trends were independent of LAB concentration but potentially dependent on species or strain specific thermotolerance and genomic differences. Interestingly, the temperature-responsive FL profile of LAB samples diverged markedly from conventional dsDNA melting curves. While standard dsDNA analysis shows monotonic FL reduction with rising temperature (Li et al., 2024; Sun et al., 2024), our LAB model demonstrated biphasic behavior: an initial FL enhancement phase (37–68 °C) followed by signal attenuation. We attributed this distinctive pattern to two sequential thermal transitions: biomembrane permeabilization phase and dsDNA denaturation phase. In biomembrane permeabilization phase (37–68 °C), progressive biomembrane disruption (Figure 2a, gray region) facilitated SYTO 9 penetration into LAB cells, amplifying fluorescence. In dsDNA denaturation phase (>68 °C), the subsequent heating melted chromosomal DNA in nonviable cells (Figure 2a, blue region), causing FL decline as dsDNA transitioned to ssDNA.

Figure 2c exhibits the derivative FL reporter of melting curves, which identified three characteristic temperatures named semilethal temperature (Ts), total lethal temperature (Td), and DNA melting temperature (Tm). We believed that at Ts point, about 50% LAB cells would die, and at Td point all the LAB cells were dead. When the temperature continued to rise to Tm point, about 50% dsDNA would melt. When the dead LAB was subjected to heating again, no biomembrane permeabilization phases were observed as shown in Figure 2d. Therefore, the two sequential thermal transitions together shaped the melting curves of the live LAB. Good reproducibility of these patterns was demonstrated through quintuplicate independent experiments (Figures 2e–h). However, when the LAB concentrations were lower than 10^6^ CFU/mL, the FL signal was not enough for drawing the melting curves (Figures 2e,f). For the quintuplicate independent experiments of *S. thermophilus*, significant differences between Ts, Td, and Tm were observed, and no intra-experiment variations were observed (Figure 2i). Therefore the three characteristic temperatures had the potential to be used for LAB classification. Plate cultivation was carried out to further confirmed the two sequential thermal transitions, as shown in Figures 2j,k. About half of the LAB died when the temperature reached 60 °C (about Ts point of Figures 2c,g); all the LAB died when the temperature reached 70 °C (about Td point of Figures 2c,g), where the FL reporter in Figures 2a,b increased to a maximum value. The results of *L. bulgaricus* and *L. casei* were similar to that of *S. thermophilus*, as shown in Supplementary Figures S1, S2. However, the three LAB strains exhibited differences in detailed melting curve and characteristic temperatures. Therefore the composite “melting curve” simultaneously reports membrane integrity loss (LAB died) and nucleic acid denaturation, providing a multiple-parameter thermal signature for LAB classification.

### 3.4 Classification of the lactic acid bacteria through multivariate dimensionality reduction of the melting curves

**Figure 3:**
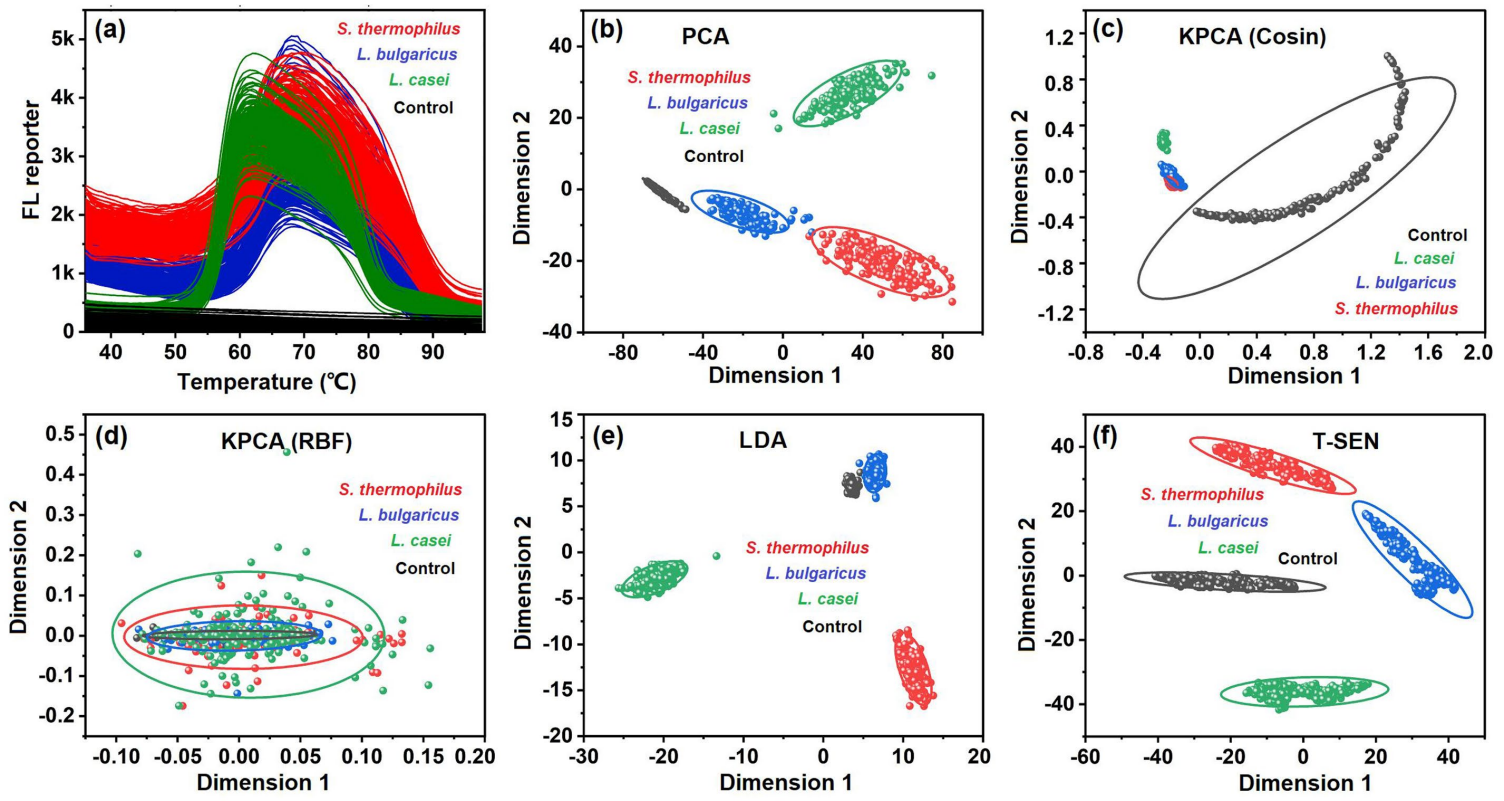
Classification of the LAB through multivariate dimensionality reduction of the melting curves. **(a)** Original melting curves for the different LAB strains, with over 200 repetitions. **(b–f)** Two-dimensional scatter plot for the melting curves, which were subjected to multivariate dimensionality reduction by PCA **(b)**, KPCA with Cosin kernel **(c)**, KPCA with RBF kernel **(d)**, LDA **(e)**, and T-SNE **(f)**. The circle represents the 95% confidence interval.

To visualize the features of different LAB strains, the Ts, Td, and Tm were extracted from the melting curves and plotted to a three dimensional scatter plot as shown in Supplementary Figure S3a. Obviously, the three LAB strains *S. thermophilus*, *L. bulgaricus*, and *L. casei* were clearly differentiated from each other. However, the absence of any one of the three parameters precludes the classification of these LAB (Supplementary Figures S3b–S3d), indicating the absolute necessity of both the biomembrane permeabilization phase and dsDNA denaturation phase for LAB classification. More simply, the melting curves could be directly converted into two-dimensional scatter plots through multivariate dimensionality reduction, as shown in Figure 3. The algorithms, principal component analysis (PCA), kernel principal component analysis (KPCA, Cosin and RBF kernels), linear discriminant analysis (LDA), and *t*-distributed stochastic neighbor embedding (T-SNE) were applied in the dimensionality reduction. For every LAB strain under investigation, over 200 individual melting curves were analyzed, with each curve corresponding to a unique LAB colony (Figure 3A). The visualization results (Figures 5b–f) demonstrated clear spatial segregation among data points representing different LAB strains following dimensionality reduction. Comparative evaluation revealed *t*-SNE’s superior performance in achieving complete discrimination between the three LAB strains. Consequently, this algorithm was selected for the subsequent analysis of melting curve patterns and LAB classification purposes.

### 3.5 Simultaneous quantification and classification of the lactic acid bacteria

**Figure 4:**
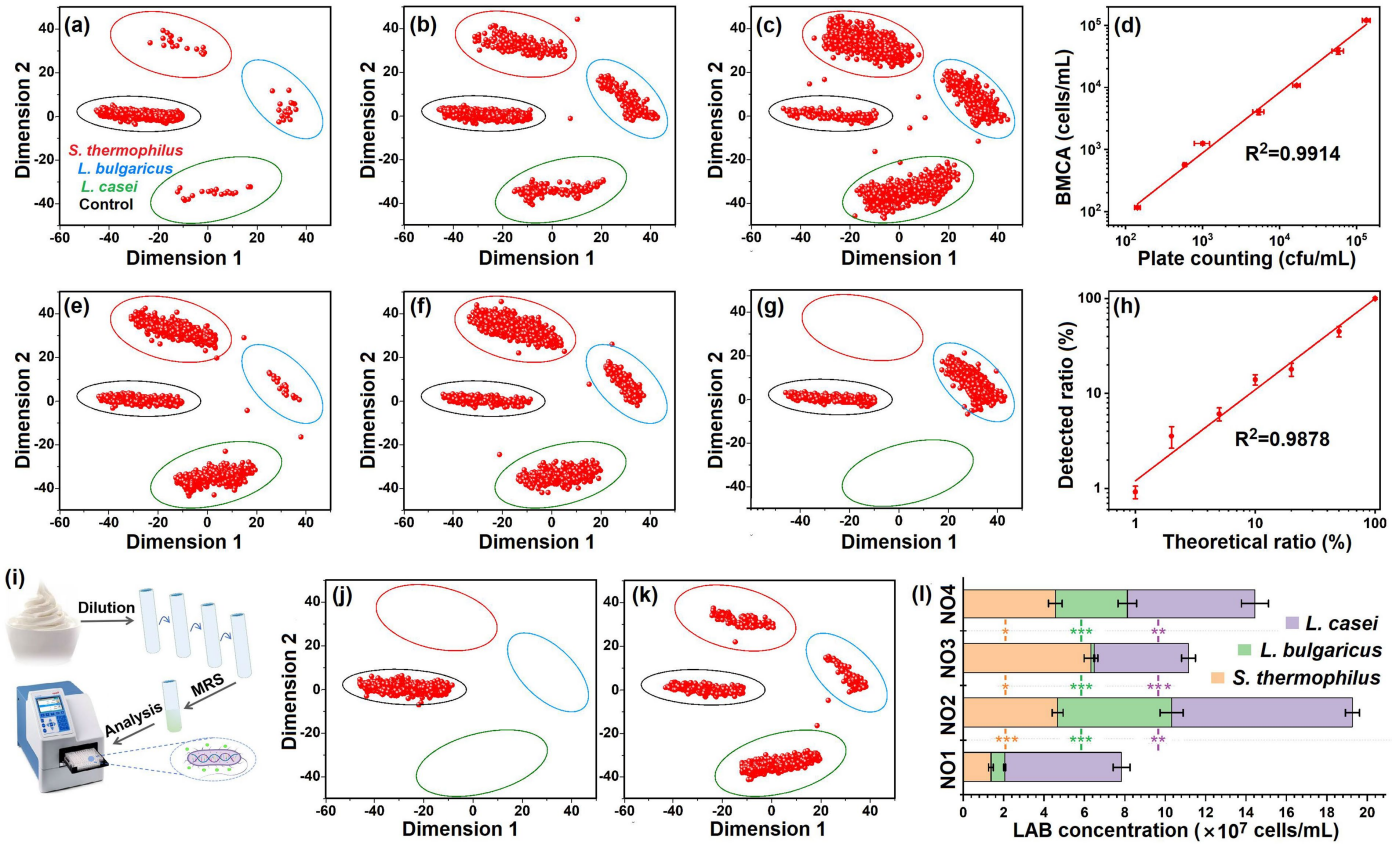
Simultaneous quantification and classification of the LAB. **(a–c)** Representative scatter plot after dimension reduction of the melting curves for UHT milk samples spiked with LAB of different concentrations. **(d)** The relationship of the detected total LAB between the BMCA and plate counting methods. The ratio of *S. thermophilus*, *L. bulgaricus*, and *L. casei* was 1 : 1 : 1, and the added total LAB ranged from 120 CFU/mL to 1 × 10^5^ CFU/mL. **(e–g)** Representative scatter plot after dimension reduction of the melting curves for UHT milk samples spiked with LAB of different inter-species ratios. The total LAB was 1 × 10^4^ CFU/mL, with *S. thermophilus* ratio ranging from 1 to 100%. **(h)** The relationship between the detected LAB ratio and theoretical ratio. **(i)** Pretreatment of the yogurt samples. **(j)** Quantification and classification of the LAB in sterilized yogurt. **(k)** Quantification and classification of the LAB in yogurt (NO1 sample) claimed to have live LAB. **(l)** Comparison in *S. thermophilus*, *L. bulgaricus*, and *L. casei* counts between different yogurt bands. Five independent experiments were conducted, and data are represented as mean ± SD (*n* = 5). The difference was significant, as indicated by one asterisk for *p* < 0.05, two asterisks for *p* < 0.01, and three asterisks for *p* < 0.001.

To simulate yogurt composition, Ultra-high temperature sterilized (UHT) milk was inoculated with three representative LAB strains: *S. thermophilus*, *L. bulgaricus*, and *L. casei*. This tri-strain combination was selected as the model system due to its prevalence in Chinese yogurt products. We firstly evaluated the method’s capability for concurrent LAB quantification and differentiation. Prior to milk inoculation, LAB cultures were grown to log phase in MRS medium to ensure viability and culturability. The single-cell derived LAB suspensions were then analyzed via BMCA. Post-dimensionality reduction, the scatter plots (Figures 4a–c) revealed four distinct clusters: one control group and three strain-specific populations. A linear relationship was observed between LAB-positive signals and spiked concentrations within a fixed volume, enabling direct quantification by scaling cell counts to sample volume. Method validation against plate counting demonstrated strong agreement (*R*^2^ = 0.9914, Figure 4d). The as-developed BMCA-based method was further tested for the analysis of targeted LAB ratio in UHT milk. The UHT milk samples spiked with three LAB strains of different *L. bulgaricus* ratios were subjected to BMCA, as shown in Figures 4e–h. An excellent correlation between the BMCA measured *L. bulgaricus* ratios and theoretical ratios was obtained with *R*^2^ of 9,878. Hence, the BMCA could achieve the simultaneous quantification and classification of the LAB.

In Walmart and other supermarkets in China, there are variety of yogurts that claim containing active LAB of multi-species and with related health benefits. According to the national standard (GB 19302-2010, China), the claimed live LAB count should be higher than 1 × 10^6^ CFU/mL. To assess these claims, the study applied the BMCA technique to quantify and identify LAB in store-bought samples. Initial processing involved serial dilution of yogurt to achieve LAB concentrations <10^2^ CFU/mL. Subsequently, 5 μL aliquots of the diluted suspension were transferred to PCR tubes preloaded with SYTO 9 and MRS broth for culturing and subsequent melting curve analysis (Figure 4i). This workflow ensured each reaction vessel contained ≤1 viable LAB cell for precise detection. For sterilized yogurt, no live LAB cells were detected (Figure 4j). The three LAB strains were indeed identified by BMCA in the yogurt claimed with live *L. casei*, *L. bulgaricus*, and *S. thermophilus* (Figure 4k). We further compared yogurts of four different bands named NO1, NO2, NO3, and NO4. All of them claimed to have the above three live LAB strains. As shown in Figure 4l, although the total LAB of the four yogurts varied from 8 × 10^7^ to 20 × 10^7^ CFU/mL, all meeting the standard of ≥1 × 10^6^ CFU/mL. Significant variations in inter-species ratio also occurred among different brands. For instance, in NO3 yogurt, *S. thermophilus* was the most abundant while in other yogurt *L. casei* showed relatively high abundance. Additionally, it was found that the live *L. bulgaricus* (2 × 10^5^) in NO3 yogurt was lower than the standard, and thus its claim to have functional live *L. bulgaricus* was arguable.

### 3.6 Thermotolerance assessment of live lactic acid bacteria using BMCA

**Figure 5:**
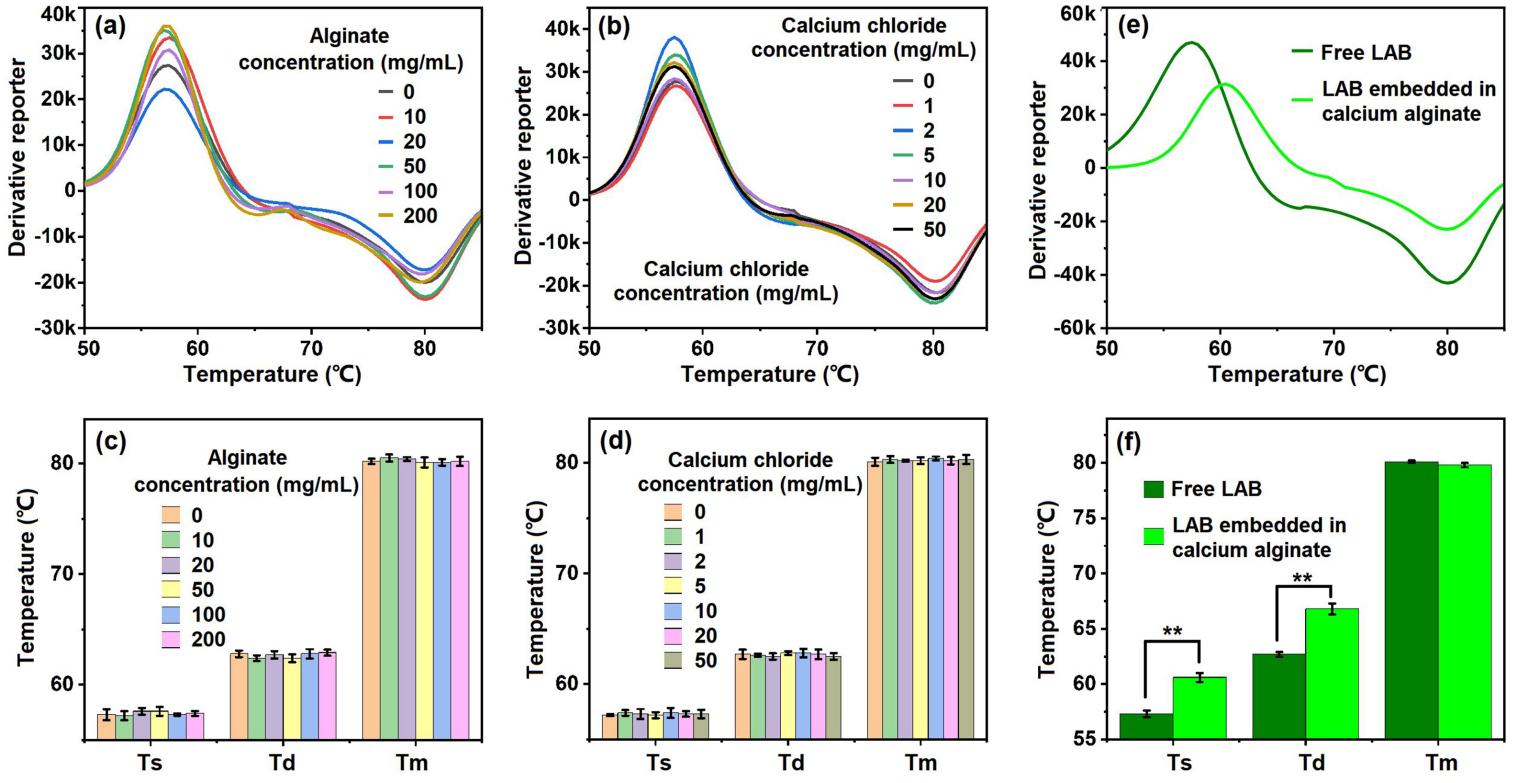
Thermo-tolerance assessment of live LAB (took *L. casei* as a model) by using the BMCA. **(a)** Effect of alginate on the melting curve of the LAB. **(b)** Effect of calcium chloride on the melting curve of the LAB. **(c,d)** Comparison in Ts, Td, and Tm values of the LAB in the presence of alginate **(c)** and calcium chloride **(d)**. **(e)** Melting curves of free LAB and LAB embedded in calcium alginate gels. **(f)** Comparison in Ts, Td, and Tm values of the free LAB and LAB embedded in calcium alginate gels. Five independent experiments were conducted, and data are represented as mean ± SD (*n* = 5). The difference was significant, as indicated by one asterisk for *p* < 0.05 and two asterisks for *p* < 0.01.

Food products distributed across regions inevitably undergo environmental stresses such as high temperatures, elevated pressure, and UV radiation, thereby accelerating spoilage (Shima et al., 2010; Mbye et al., 2020; Wu Z. et al., 2025). Most LAB strains are sensitive to thermal change, necessitating refrigerated storage to maintain LAB viability throughout the storage period for conventional live-probiotic foods and drugs. Such stringent storage requirements substantially increase logistical and preservation costs. Recent efforts have sought to enhance probiotic stress resistance through encapsulation or surface modification strategies (Thi-Tho et al., 2022; Kwon et al., 2025; Xie et al., 2025). Here, we comparatively evaluated the thermotolerance of LAB in different formulations by using BMCA to capture their characteristic temperatures. The edible calcium alginate was used to prepared encapsulated LAB. Figures 5a,b show the melting curves (exhibited as derivative reporter) of the LAB in the absence and presence of alginate (Figure 5a) and calcium chloride (Figure 5b). Neither alginate nor calcium chloride alone could changed the melting curve of the tested LAB. All the characteristic temperatures were consistent among alginate and calcium chloride of different concentrations (Figures 5c,d), indicating that they had no influence on the thermotolerance of LAB. However, when the alginate and calcium chloride were combined to encapsulated the LAB into the calcium alginate gel, the encapsulated LAB exhibited migration at characteristic temperatures (Figures 5e,f). Both Ts and Td values for the LAB embedded in calcium alginate were higher than that for free LAB. Therefore, calcium alginate gel may serve as a heat barrier to improve the thermotolerance of LAB.

## 4 Conclusion

The expanding market for probiotic fermented foods like yogurt necessitates advanced methodologies to validate product claims. In the present research, we found SYTO 9, as a dsDNA-specific dye, preferred penetrating dead LAB. Based on this, we established a BMCA method for the rapid classification of LAB. In the method, SYTO 9 coupled with a thermoregulated fluorometer were employed to concurrently track the dynamic change of the LAB viability and dsDNA, drawing species-specific melting curves. The curves were attributed by two sequential thermal transitions: biomembrane permeabilization phase and dsDNA denaturation phase. In the first phase, progressive biomembrane disruption facilitated SYTO 9 penetration into LAB cells, increasing FL. In the second phase, the subsequent heating melted dsDNA in nonviable cells, causing FL decline as dsDNA transitioned to ssDNA. Unambiguous discrimination of the three model LAB through multivariate dimensionality reduction of the melting curves was demonstrated. The novel approach effectively enabled LAB identification and measurement in yogurt samples without requiring antibody-based techniques. However, the BMCA was conducted only on three relatively easy-to-culture LAB strains, while there are over a 100 species of LAB used in industrial applications. We anticipate that future research will focus on establishing a standardized library of bacterial melting curves for LAB. By querying this library, the BMCA method can be applied to identify the species in any LAB product, including pharmaceuticals, yogurts, and probiotic preparations. Additionally, the BMCA method can also be employed to evaluate thermal stability of LAB in formulations.

## Data Availability

The original contributions presented in the study are included in the article/Supplementary material, further inquiries can be directed to the corresponding authors.

## Footnotes

1
https://www.spsspro.com/
