# Supplementary material for: Classification, quantification, and thermotolerance assessment of lactic acid bacteria in yogurt using bacterial melting curve analysis
Source: Front Microbiol. 2026 Mar 4;17:1751797. doi: 10.3389/fmicb.2026.1751797 (PMC12996188; doi:10.3389/fmicb.2026.1751797)
Supplement: Supplementary file 1 [file Data_Sheet_1.docx]

**Supporting Information for**

**Classification, quantification, and thermotolerance assessment of live lactic acid bacteria using bacterial melting curve analysis**

This Supporting Information includes Supplementary Figure S1, Figure S2, and Figure S3.

**
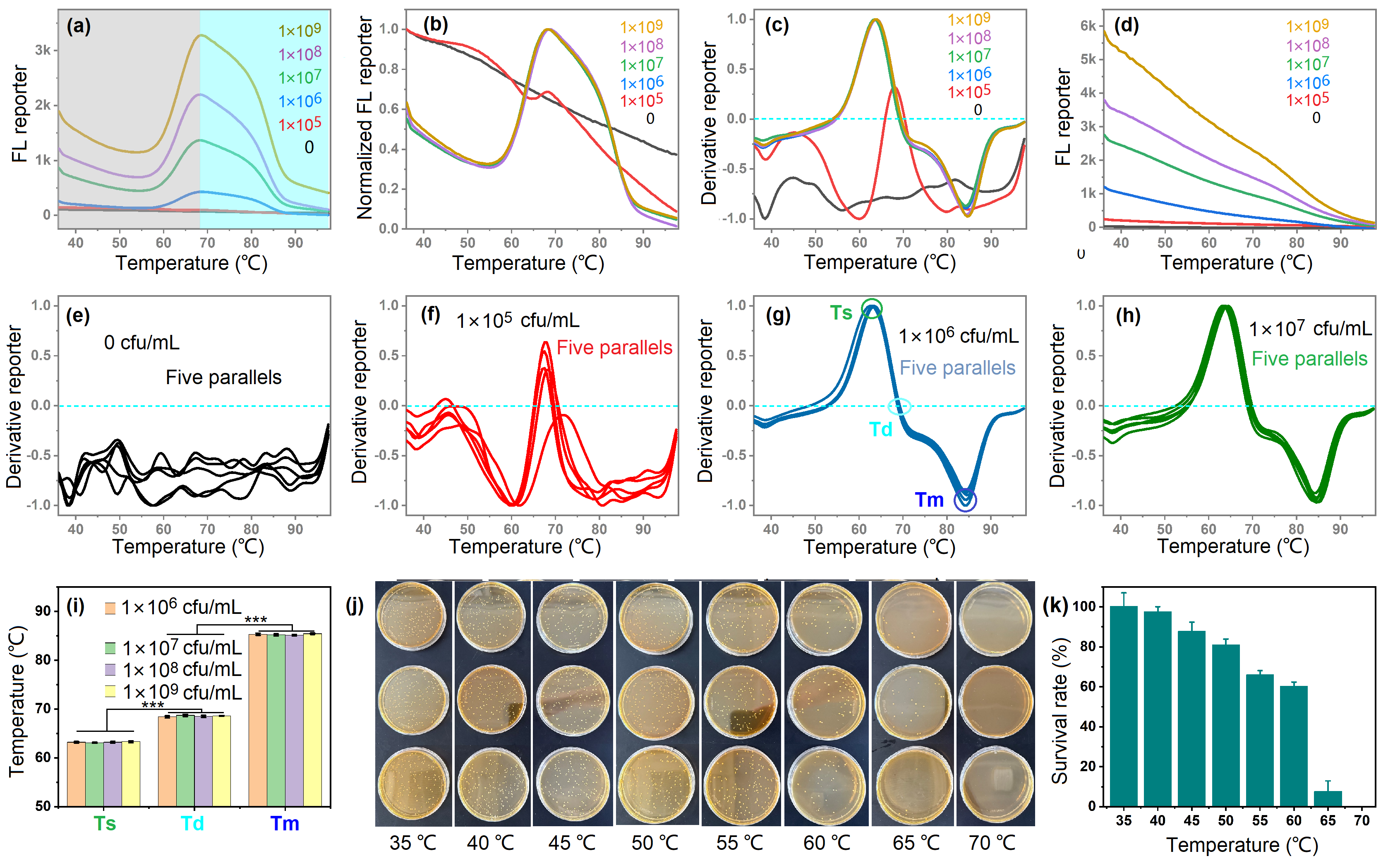
**

**Supplementary Figure S1.** Two sequential thermal transitions shaped the bacterial melting curves. (a) Melting curves for live L. bulgaricus of different concentrations, ranging from 0 to 1×10^9^ cfu/mL. (b-c) Normalization (b) and derivation (c) of the FL reporter for the above samples. (d) Melting curves for dead L. bulgaricus of different concentrations. (e-h) Melting curves for repeated experiments of live L. bulgaricus of different concentrations. (i) Statistic analysis of semilethal temperature (Ts), total lethal temperature (Td), and DNA melting temperature (Tm) of the above samples. (j) Influence of temperature on the viability of L. bulgaricus evaluated by plate counting. (k) Survival rate of the LAB calculated from the plate counting results.


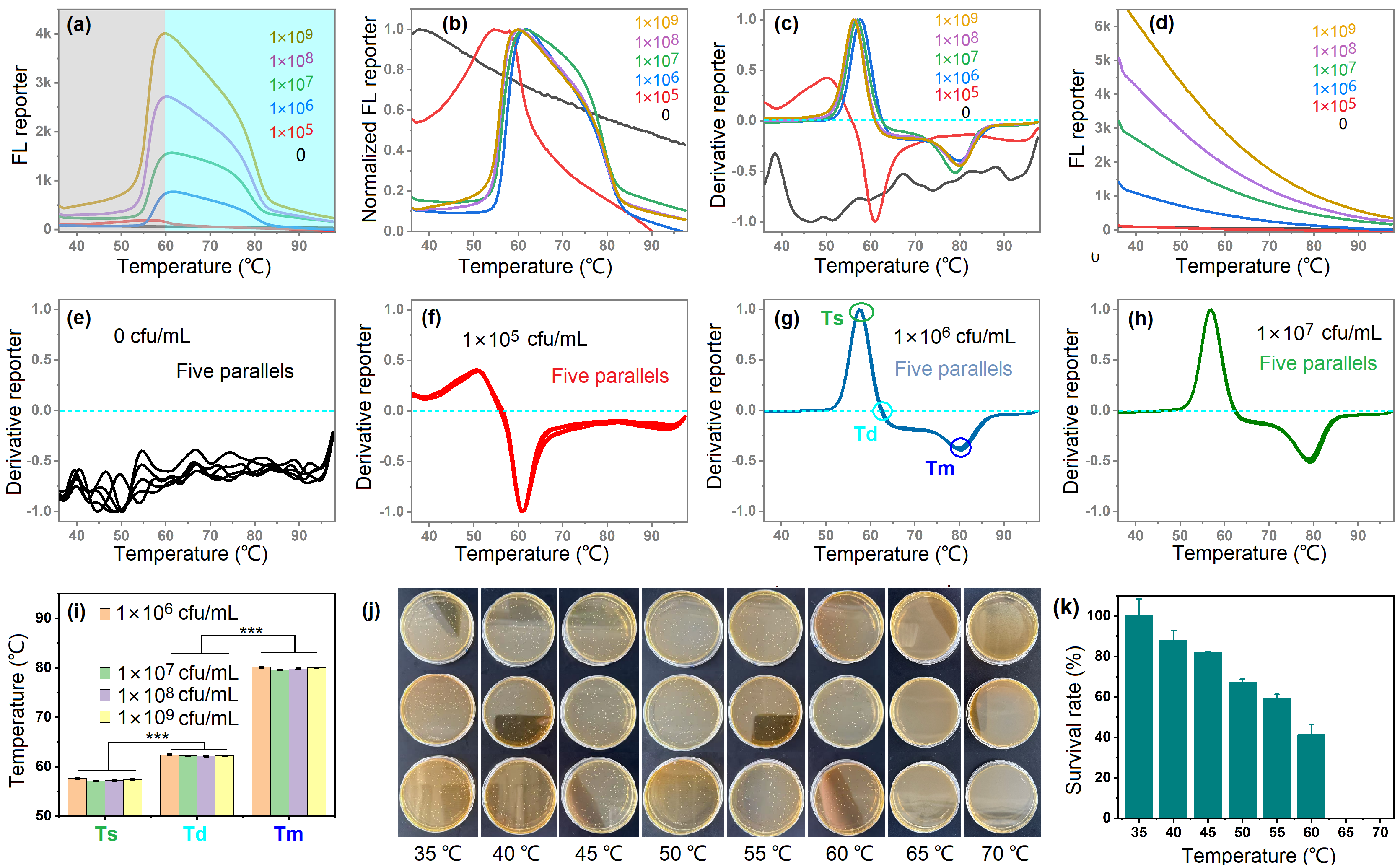


**Supplementary Figure S2**. Two sequential thermal transitions shaped the bacterial melting curves. (a) Melting curves for live L. casei of different concentrations, ranging from 0 to 1×10^9^ cfu/mL. (b-c) Normalization (b) and derivation (c) of the FL reporter for the above samples. (d) Melting curves for dead L. casei of different concentrations. (e-h) Melting curves for repeated experiments of live S. thermophilus of different concentrations. (i) Statistic analysis of semilethal temperature (Ts), total lethal temperature (Td), and DNA melting temperature (Tm) of the above samples. (j) Influence of temperature on the viability of L. casei evaluated by plate counting. (k) Survival rate of the LAB calculated from the plate counting results.


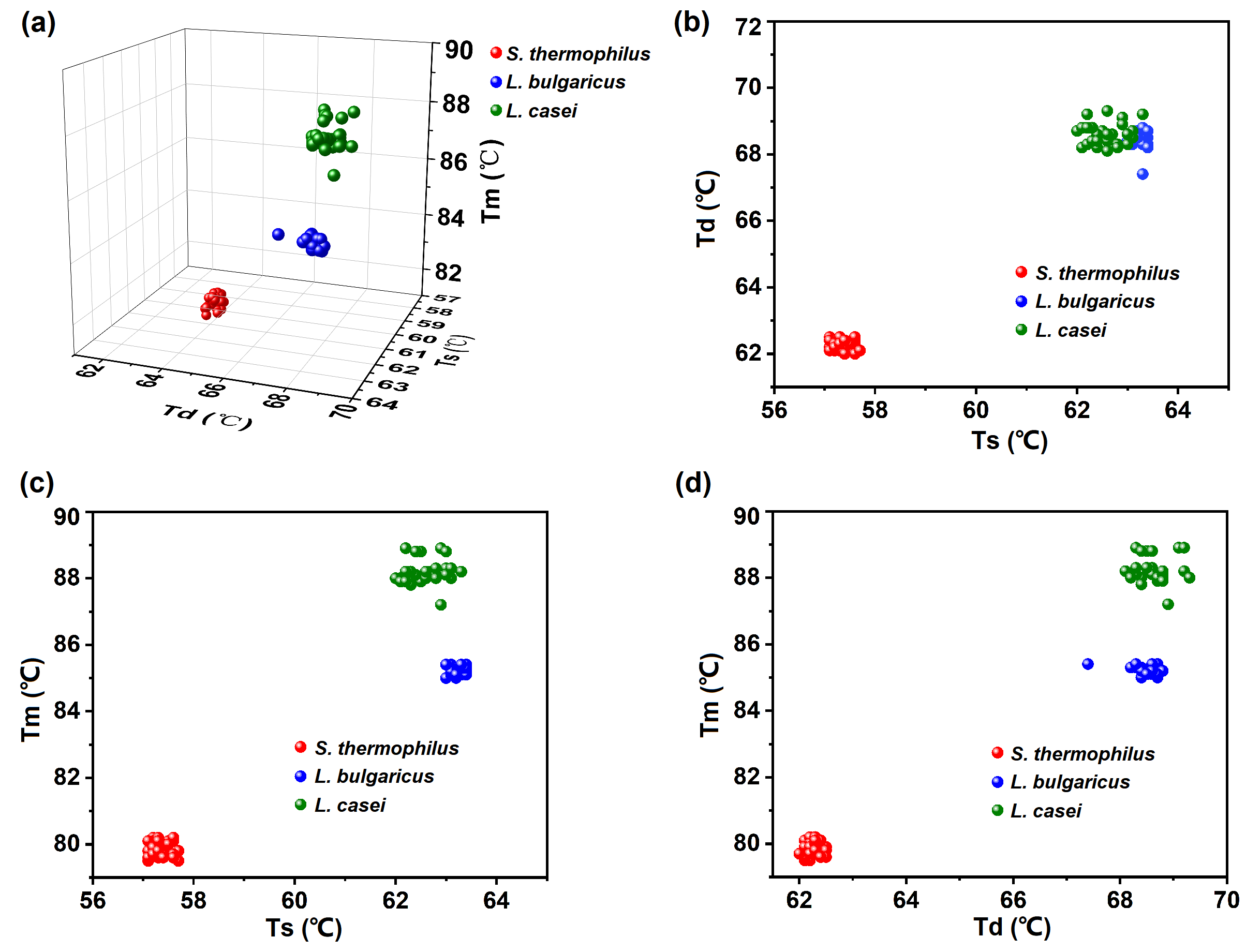


**Supplementary Figure S3**. Distribution of different LAB strains in scatter diagrams. (a) Scatter diagram plotted by Ts, Td, and Tm. (b) Scatter diagram plotted by Ts and Td. (c) Scatter diagram plotted by Ts and Tm. (d) Scatter diagram plotted by Td and Tm.
